# Adherence to Canadian 24-Hour Movement Guidelines among infants and associations with development: a longitudinal study

**DOI:** 10.1186/s12966-022-01397-8

**Published:** 2022-12-15

**Authors:** Valerie Carson, Zhiguang Zhang, Madison Predy, Lesley Pritchard, Kylie D. Hesketh

**Affiliations:** 1 Faculty of Kinesiology, Sport, and Recreation, University of Alberta, Edmonton, AB Canada; 2 Faculty of Rehabilitation Medicine, University of Alberta, Edmonton, AB Canada; 3 Institute for Physical Activity and Nutrition, Deakin University, Geelong, Vic Australia

**Keywords:** Infants, Tummy time, Sedentary behaviour, Sleep, Guidelines, Development

## Abstract

**Background:**

To examine: 1) longitudinal adherence to the Canadian 24-Hour Movement Guidelines in a sample of infants and 2) associations between adherence to the guidelines over time and development.

**Methods:**

Participants were 250 parent-infant dyads from the Early Movers project in Edmonton, Alberta. At 2, 4, and 6 months of age, physical activity, sedentary behaviour, sleep, and development were measured with a parental questionnaire that included items from the Ages & Stages Questionnaire (ASQ-3). Parents also reported the dates six major gross motor milestones were acquired during the first 18 months of life according to World Health Organization criteria. In a sub-sample (*n* = 93), movement behaviours were also measured with a time-use diary at 2, 4, and 6 months and gross motor development was measured by a physiotherapist using the Alberta Infant Motor Scale (AIMS) at 6 months. Guideline adherence was defined as: 1) ≥ 30 min/day of tummy time, 2) no screen time, some reading time, no restrained bouts > 1 h (time-use diary only), and 3) 14–17 h (2 months) or 12–16 h (4 and 6 months) of sleep per 24-h period. Generalized estimating equations were conducted as well as linear mixed models and linear regression models that adjusted for demographic characteristics.

**Results:**

Few infants met the guidelines at all time-points (questionnaire: 2%; time-use diary: 0%). Infants that met a recommendation at 2 months, compared to those that did not, were 1.8–8.2 times more likely to meet that recommendation at subsequent time-points. Meeting more recommendations across time-points, according to both measures, was associated with a higher mean ASQ-3 gross motor score. Each additional time-point of tummy time recommendation adherence (questionnaire-measured) was associated with a 5–11-day earlier acquisition of independent sitting, crawling, and independent standing milestones. In the sub-sample, each additional time-point of guideline adherence was associated with a 16% higher AIMS score at 6 months.

**Conclusions:**

Guideline adherence was low across the first 6 months of infancy. Overall, meeting more recommendations over this period appeared important for gross motor development. Parents and caregivers should be targeted as early as possible with guideline dissemination and activation strategies to promote healthy infant development.

**Supplementary Information:**

The online version contains supplementary material available at 10.1186/s12966-022-01397-8.

## Background

The Canadian 24-Hour Movement Guidelines for the Early Years (0–4 years) were released in 2017 providing guidance to various stakeholders who play a role in facilitating and supporting healthy development during this critical developmental period [1]. In line with similar Canadian guidelines in other age groups, including school-aged children and youth (5–17 years) [2] and adults (18 + years), recommendations are provided for three movement behaviours: physical activity, sedentary behaviour, and sleep [3]. Other countries and organizations have adopted the Canadian guidelines [4]. In particular, for the early years age group, the World Health Organization (WHO) released guidelines in 2019, primarily based on the work completed in Canada [5]. Given the vast developmental differences in the first four years of life [6], these national and international guidelines include distinct recommendations for infants (< 1 year), toddlers (1–2 years) and preschoolers (3–4 years) [1].

One important outcome of guideline development is the creation of benchmarks that can be used in population health surveillance work to determine what proportion of the population is at risk for suboptimal health [1]. Surveillance work conducted in Canada, as part of the 2017 guideline release, suggested a large proportion of toddlers and preschoolers may be at increased health risk because less than 15% met the 24-Hour Movement Guidelines [7, 8]. Internationally, a systematic review and meta-analysis across 26 studies from 14 different countries that was published in 2022 found only 11% of preschoolers met the overall guidelines [9]. Of note, this surveillance evidence is primarily based on cross-sectional study designs, with only 3 included studies [10–12] using a longitudinal study design [9]. To date, in Canada, surveillance evidence for the 24- Hour Movement Guidelines does not exist for the infant group. Internationally, only one study from Australia, which used a cross-sectional study design, has focused on guideline surveillance in this age group [13]. Similar to the majority of surveillance work among toddlers and preschoolers in Canada [7, 8, 14], data used in this Australian study were collected prior to the release of the guidelines [13], which makes it difficult to draw conclusions regarding guideline adherence.

The creation of benchmarks as part of the guideline development process can also foster future research that aims to understand the associations of meeting individual recommendations and overall guidelines with a variety of health outcomes across age groups and countries. A systematic review published in 2020 [15] on the relationship between meeting the 24-Hour Movement Guidelines and health indicators across the lifespan, only found one study in infants [13] compared to two studies in toddlers [8, 16] and nine studies in preschoolers [7, 14, 17–22]. To our knowledge, no study internationally has examined the associations between guideline adherence over time and various domains of development in infants. In an effort to address evidence gaps, the primary objectives of this study were to examine the: 1) longitudinal adherence to the Canadian 24-Hour Movement Guidelines in a sample of infants and 2) associations between adherence to the guidelines over time and development across multiple domains.

## Methods

### Study design and participants

This study includes data from the Early Movers project, which used a longitudinal study design. Participants were parents/guardians (parents thereafter) and their infants who were recruited while attending routine 2-month immunization appointments at one of five Public Health Centres in Edmonton, Canada that serve diverse populations. The provincial health authority (Alberta Health Services) helped to facilitate recruitment in the waiting rooms of these centres. The uptake of 2-month immunizations in the population of infants residing in Edmonton, Canada is approximately 85% (Personal Communication, Alberta Health Services, May 8, 2017). Recruitment took place between March, 2018 and November, 2019. Eligibility criteria for the Early Movers project has been previously reported [23, 24].

It is often challenging in movement behaviour research to use measures with high precision in large and diverse samples [25, 26]. The Early Movers project tried to address this challenge by enrolling participants in different groups or sub-studies based on measurement precision and burden. Further details on the group structure of the Early Movers project has been previously published [24]. Briefly, for the purpose of this study, all participants were enrolled in the main study, which included the completion of lower-burden questionnaire measures. Also, a sub-sample of participants agreed to be enrolled in a time-use diary sub-study that included additional measures with higher precision and burden (i.e., time-use diaries, physical therapist- assessed gross motor development). A total of 808 families were recruited for the Early Movers project. Ethics approval was obtained from the University of Alberta Research Ethics Board (Project # 00,078,438). Written informed consent was obtained from all participating parents. Details on the *apriori* power calculations for the main study and the time-use diary sub-study have been previously published [24].

### Procedures

Contacts at each health centre informed research staff of scheduled 2-month immunization appointments. Research staff visited the waiting room when multiple appointments were scheduled and spoke to parents before or after their appointments. Families are required to stay in the waiting room for 15 min after their infant’s immunization for safety reasons. Interested and eligible parents completed a consent form, contact information form, and a parental questionnaire at the health centre using the secure web application REDCap [27] or on a hard paper copy. Parents were also given a gross motor milestone questionnaire to take home. Participants were then emailed a survey link to the follow-up questionnaires via REDCap or were mailed a hard copy of the questionnaires to be returned via mail when their infants turned 4 and 6 months of age. Next, participants were contacted monthly regarding the achievement of gross motor milestones until their child had reached all the milestones or was 18 months of age [28].

Parents who agreed at the immunization appointment to participate in the time-use diary sub-study were also asked to complete a 3-day/night time-use diary when their infant was 2, 4, and 6 months of age. Participants received a hard copy of the diary at the immunization appointment, along with verbal and written instructions. Subsequent time-use diaries were mailed to participants and returned via mail when infants turned 4 and 6 months of age. Additionally, when infants were between the ages of 6 months 0 days and 6 months 7 days, a physical therapist made a home visit to assess infants’ gross motor development. Gift cards were mailed to participants upon completion of data collection. Participants who enrolled in the main study only were eligible for a gift card of $25 CAD in value. Participants who enrolled in the main study and the time-use diary sub-study were eligible for a gift card of $35 CAD in value, given the extra burden of measures associated with the sub-study. Pro-rated gift card amounts were provided if participants withdrew early from the study.

### Measures

#### Questionnaire-measured movement behaviours

Infant movement behaviours were measured using the parental questionnaire when infants were 2, 4, and 6 months of age. For physical activity, tummy time was measured with one question asking parents to report the typical time per day their child spends awake on their stomach when they are free to move. For sedentary behaviour, reading time was measured with one question asking parents to report the typical time per day their child spends reading/looking at books with the parent or another child/adult. Additionally, screen time was measured with two separate questions asking parents to report the typical time per day their child spends: 1) watching/looking at the television and 2) watching/looking at a cell phone/tablet. Responses were summed across screen time questions. Finally, for sleep, sleep time was measured with two separate questions asking parents to report the typical time their child: 1) usually sleeps in total per night at the moment (not including time spent feeding) and 2) naps in total during the day at the moment. Responses were summed across sleep questions. Detailed information on the psychometric properties of the questionnaire-measured movement behaviours in the Early Movers project have been previously reported [23, 24]. Briefly, these movement behaviour questions have been adapted from previous studies, where test re-test reliability has been reported (Intraclass correlation coefficient: ICC = 0.20 to 0.86) [29, 30]. Within sub-samples of the Early Movers participants, concurrent validity for the tummy time measure (against an accelerometer; r_s_ = 0.60, *p* < 0.05) and all movement behaviour measures (against the time-use diary described in the next section; *r*_*s*_ = 0.30–0.56; *p* < 0.05) have also been reported [24, 31].

#### Time-use diary-measured movement behaviours

Infant movement behaviours were measured using the time-use diary when infants were 2, 4, and 6 months of age. Over three 24 h periods, parents recorded in 5 min intervals their infant’s main activity from a list of 17 options and their infant’s position from a list of 10 options. For physical activity, tummy time included the average time across valid days that infants spent on their front/tummy or army/commando crawling (i.e., infant on tummy with some movement; 6 months: *n* = 7) position while awake. For sedentary behaviour, screen time included the average time across valid days that infants’ main activity was TV and/or cell phone/tablet. Additionally, reading time included the average time across valid days that infants’ main activity was reading (by an adult or another child). Finally, restrained time bouts were the average time across valid days that infants’ main activity was stroller ride, car ride, carrier, indoor swing, or other restricted activity (e.g., high chair, car seat) for a consecutive period greater than 1 h (while awake). For sleep, sleep time included the average time across valid days that infants’ main activity was sleeping. Further details regarding this time-use diary, including psychometric properties, have been described in detail elsewhere [31]. Briefly, the time-use diary was adapted from a previous study in adults, where test re-test reliability (ICC of 0.50 to 0.55) was reported [32]. Within a sub-sample of the Early Movers project (*n* = 26), concurrent validity for the tummy time measure (against an accelerometer; *r*_*s*_ = 0.80, *p* < 0.05) has also been previously reported [31].

#### Guideline adherence

Both the questionnaire-measured and time-use diary-measured movement behaviour variables were categorized as meeting versus not meeting the infant recommendations within the Canadian 24-Hour Movement Guidelines for the Early Years [1]. Specifically, to meet the physical activity recommendation, infants had to engage in at least 30 min of tummy time per day. To meet the sedentary behaviour recommendation, two definitions were developed. For definition 1, infants had to engage in no screen time and some reading time. For definition 2, which could only be assessed in the time-use diary sub-sample, infants had to engage in no screen time, some reading time, and no restrained bouts greater than 1 h. To meet the sleep recommendation, infants had to sleep 14 to 17 h per 24-h period when they were 2 months of age and 12 to 16 h per 24-h period when they were 4 and 6 months of age. Meeting the overall guidelines was defined as meeting the physical activity, sedentary behaviour, and sleep recommendations.

#### Development

The Early Movers project included several measures of development. Communication, fine motor, gross motor, personal-social, problem solving and total development were measured at 2, 4, and 6 months of age with the Ages and Stages Questionnaire (ASQ-3) [33]. Specifically, at each time point, 30 items, specific to the age group, were included in the parental questionnaire with three response options (yes, sometimes, not yet). Each area of development was given a score between 0 and 60 and these scores were summed for the total development score, with higher scores indicating more advanced development [33]. Further details on the scoring of the ASQ-3 can be found elsewhere [24]. Validity for the ASQ-3 tool has previously been reported (Criterion validity against Battelle Developmental Inventory-II: Percent agreement at 2 months = 100%; 4 months = 83.3%; 6 months = 85.7%) [33].

The dates children acquired six gross motor milestones (independent sitting, hands and knees crawling, assisted standing, assisted walking, independent standing, and independent walking) in the first 18 months of life were reported by parents in a separate questionnaire. The questionnaire included detailed instructions and pictures from the World Health Organization (WHO) on how to determine if the milestones were achieved [28]. Parents also recorded whether the dates provided was exact or approximate. Further details on how milestone data were cleaned for the Early Movers project has been previously published [24]. Children were only followed up to 18 months as World Health Organization reference data indicates that 99% of children typically acquire these six milestones by this age [28].

In the time-use diary sub-sample, gross motor development was also directly observed by a physical therapist when infants were 6 months of age using the Alberta Infant Motor Scale (AIMS) [34]. Specifically, a total of 58 items were scored across four postural positions (Prone: 0–21, Supine: 0–9, Sitting: 0–12, Standing: 0–16), and a total score was calculated by summing the prone, supine, sitting and standing subscale scores [34]. Additionally, infants were assigned a percentile score between 0 and 100 based on their total score and age. The percentile score is based on normative data of 2,220 infants from Alberta in 1990–1992. Higher AIMS scores indicate more advanced gross motor development. Reliability (test–retest: *r* = 0.96–0.99) and validity (Concurrent validity: Bayley Scales of Infant Development motor scales: *r* = 0.98 and Peabody Development Motor Scales: *r* = 0.97) have previously been reported for the AIMS tool [34].

#### Covariates

Several infant and parental demographic characteristics that were measured in the parental questionnaires were considered as covariates based on previous research [35, 36]. Infant age, expressed as days, was calculated at each time point based on the date of questionnaire completion and the birth date reported at baseline. Non-parental care time (hours per week) was also reported at each time point. Infant sex, race/ethnicity and number of siblings were reported at baseline only. Infant sex had two response options (male, female), and response options for race/ethnicity (Caucasian, other) and number of siblings (zero, one, two or more) were collapsed from the original scales due to frequency distributions. Parental age, expressed as years, was calculated at each time point in conjunction with the infant age calculation. Mean imputation was performed for missing parental age data at baseline for one participant. Parental martial status (married/living common-law, not married/ living common-law), education (below bachelor level, bachelor’s degree, above bachelor level), and country of birth (Canada, other) were reported at baseline only, and were collapsed from the original scales due to frequency distributions.

### Statistical analysis

Statistical analyses were performed using SAS version 9.4 (SAS Institute Inc., Cary, NC) and SPSS version 26.0 (SPSS Inc., Chicago, IL, USA). Descriptive statistics were calculated for demographic characteristics and for guideline adherence. To address objective 1, generalized estimating equations (GEE) were performed to calculate exponentiated longitudinal tracking coefficients (odds ratio [OR]). Specifically, individual recommendation or overall guideline adherence (meeting vs. not meeting) at 2 months was regressed on the corresponding longitudinal individual recommendation or overall guideline adherence from 4 to 6 months. Time point (4 and 6 months) was included in the model as a within-subject variable and an unstructured correlation structure was used for all models.

To address objective 2, continuous adherence variables were calculated based on the number of recommendations met across the three time-points. For individual recommendation adherence variables (i.e., tummy time, screen time, reading time, restrained time bouts, sedentary behaviour definition 1 [no screen time, some reading time], sedentary behaviour definition 2 [no screen time, some reading time, no restrained bouts ≥ 1 h], sleep time) the possible range was 0 to 3. Similarly, for overall guideline adherence variables (i.e., physical activity + sedentary behaviour definition 1 + sleep or physical activity + sedentary behaviour definition 2 + sleep) the possible range was also 0 to 3. A final continuous guideline adherence variable was calculated based on the total number of recommendations. The possible range for this variable was 0 to 9, given there are three movement behaviours (i.e., physical activity, sedentary behaviour (definition 1 or 2), sleep) and three time points (2 months, 4 months, 6 months). To examine the associations of recommendation and overall guideline adherence over time with ASQ-3 outcomes over time, linear mixed models were conducted. Separate models were run for each combination of guideline adherence variable and ASQ-3 outcome variable. In all models, time was included as a repeated and fixed effect and covariates were included as fixed effects. Analyses were conducted for both the questionnaire data and time-use diary data. Assumptions for linear mixed models were checked through visual inspection of residuals and all assumptions were met. The unstandardized beta coefficient can be interpreted as the pooled within- and between-individual differences in the ASQ-3 outcome variable for each additional time a recommendation or the overall guidelines are met across the three points.

To examine the associations of recommendation and overall guideline adherence over time with gross motor milestone and AIMS outcomes, linear and logistic regression models were conducted. The analyses for the AIMS outcomes were only conducted with the time-use diary measured movement behaviours as this outcome was only measured in the time-use diary sub-study. A logistic regression model was only implemented for the AIMS stand variable because a non-normal distribution was observed for this variable when the distributions of the model residuals were visually checked. As a result, this variable was dichotomized (value = 1 [score = 2, reference group]; value = 0 [score > 2]). All other linear regression model assumptions were met across models. All covariates were included in all models, except infant age, which was excluded from gross motor milestone models, since age was the unit of the gross motor milestone variables. Sensitivity analyses was conducted examining the associations between movement behaviours over time and gross motor milestones in those where exact milestone dates were reported. Findings from the sensitivity analysis were then compared with the findings from the main analysis where exact and approximate milestone dates were included. For the linear regression models, the unstandardized beta coefficient can be interpreted as the mean difference in the gross motor milestone or AIMS outcome variable for each additional time a recommendation or the overall guidelines are met across the three points. For the logistic regression model, the odds ratio can be interpreted as the likelihood of achieving a higher AIMS stand score, compared to a lower score, for each additional time a recommendation or the overall guidelines are met across the three points.

Additional analyses were conducted to address objective 2 by calculating categorical adherence variables based on participants who consistently met or did not meet the recommendations across all three time points. These analyses were not conducted for overall guidelines adherence outcomes due to low adherence. Linear mixed models and linear and logistic regression models were repeated as described for the continuous adherence variables above. For all analyses, participants with observations for all variables of interest were included. Statistical significance was defined as *p* < 0.05 for all analyses.

## Results

Of the 808 families who were recruited for the Early Movers project, 178 were ineligible and 207 declined to participate, leaving a sample of 423 families (67% participation rate) across groups. A breakdown of the reasons for ineligibility and for declining participation have been previously reported [24]. Of the 423 eligible families that agreed to participate, 250 were included in this study. A total of 173 participants were excluded for the following reasons: no valid questionnaire-measured movement behaviour data at all three time points (*n* = 162), medical condition or delay diagnosed during the study (*n* = 4), and withdrew before providing any data (*n* = 7). The analytic questionnaire sample had significantly older infants at 4 and 6 month time-points, older parents at 2, 4, and 6 month time-points, a higher proportion of more educated parents, and a higher proportion of married or living in common-law parents, compared to the sample that did not have valid movement behaviour data at all three time points (*p* < 0.05).

A total of 195 out of 423 eligible families also agreed to participate in the time-use diary sub-study, and 94 were included in this study. A total of 101 participants were excluded for the following reasons: no valid time-use diary-measured movement behaviour data at all three time points (*n* = 83); moved to the main study only or group 1 (*n* = 14), medical condition or delay diagnosed during the study (*n* = 2), and withdrew before providing any data (*n* = 2). Of note, five of the 94 included participants did not have valid questionnaire-measured movement behaviour data at all three time points and therefore were not part of the analytic questionnaire sample for this study. With the exception of infant age at 6 months, there were no significant differences in demographic characteristics between the analytic diary sub-sample and those not having valid time-use diary measured movement behaviours at all three time-points. Specifically, the analytic sub-sample was slightly younger (185.3 days versus 188.5 days) at 6 months. The completion rate for the diaries of the 94 included participants was high at all three time-points (2 months: 99.0 ± 1.6%; 4 months: 97.8 ± 5.5%; 6 months: 96.9 ± 7.6%).

Demographic characteristics for the questionnaire and time-use diary data samples across time-points is provided in Table 1. Both samples included slightly more female infants (56%). Overall, the samples were relatively diverse. For instance, approximately two thirds of infants (37%) in the time-use diary sub-sample and approximately half (47%) in the questionnaire sample were classified by their parents as a race/ethnicity other than Caucasian. Additionally, 20% and 30% of infants in the time-use dairy sub-sample and questionnaire sample, respectively, had parents who were not born in Canada. Finally, about two thirds of the samples (questionnaire: 36%; time-use diary: 30%) had a parent with an education below a bachelor’s degree.

**Table 1:** Demographic characteristics for questionnaire and time-use diary samples at 2, 4, and 6 months of age

Demographic characteristics	Questionnaire (*n* = 250)			Time-use diary(*n* = 94)		
	2 month	4 month	6 month	2 month	4 month	6 month
Infant age (days)	66.58(5.79)	127.39(8.10)	186.40(7.14)	67.09(5.62)	126.49(7.22)^c^	185.29(5.51)^c^
Infant sex						
Male	109(43.6)	-	-	41(43.6)	-	-
Female	141(56.4)	-	-	53(56.4)	-	-
Infant race/ethnicity						
Caucasian	132(52.8)	-	-	59(62.8)	-	-
Other	118(47.2)	-	-	35(37.2)	-	-
Number of siblings						
Zero	116(46.4)	-	-	53(56.4)	-	-
One	98(39.2)	-	-	31(33.0)	-	-
Two or more	36(14.4)	-	-	10(10.6)	-	-
Non-parental care (hours)	1.95(10.10)	2.43(7.26)	2.67(6.65)	1.14(3.35)	1.69(3.90)^c^	2.07(4.51)^c^
Parental age (years)	32.62(5.03)^a^	32.56(4.85)^b^	32.69(4.78)^b^	32.29(4.22)	32.44(4.15)^d^	32.52(4.00)^d^
Parental marital status						
Married or living common-law	241(96.4)	-	-	90(95.7)	-	-
Not married or living common-law	9(3.6)	-	-	4(4.3)	-	-
Parental education						
Above bachelor’s degree	62(24.8)	-	-	25(26.6)	-	-
A bachelor’s degree	98(39.2)	-	-	41(43.6)	-	-
Below a bachelor’s degree	90(36.0)	-	-	28(29.8)	-	-
Parental country of birth						
Canada	173(69.2)	-	-	75(79.8)	-	-
Other country	77(30.8)	-	-	19(20.2)	-	-

Data presented as mean (standard deviation) for continuous variables and frequency (percentage) for categorical variables

^a^Mean imputation was performed for missing parental age at baseline (*n* = 1)

^b^ *n* = 249

^c^ *n* = 91

^d^ *n* = 90

The proportion of participants meeting individual recommendations and the overall guidelines for questionnaire sample and time-use diary sub-sample at 2, 4, and 6 months of age is displayed in Table 2. In the questionnaire sample, 40%, 17%, and 34% of infants met the physical activity, sedentary behaviour (screen time + reading time), and sleep recommendations at all three time points, respectively. In the time-use diary sub-sample, 9%, 15%, 28% of infants met the physical activity, sedentary behaviour (screen time + reading time), and sleep recommendations at all time points, respectively. However, when the restrained time recommendation was included, no infants in the time-use diary sub-sample met the sedentary behaviour recommendations (screen time + reading time + restrained time) at all time points. In terms of the overall guidelines, few infants met them at all time-points (questionnaire sample: 2%; time-use diary sub-sample: 0%).

**Table 2:** Proportion of participants meeting recommendations and overall guidelines for questionnaire and time-use diary samples at 2, 4, and 6 months of age

Recommendations	Questionnaire				Time-use diary			
	2 month	4 month	6 month	All time points	2 month	4 month	6 month	All time points
**Physical activity recommendation**								
Tummy time	128(51.2)	172(68.8)	228(91.2)	101(40.4)	17(18.1)	38(40.4)	59(62.8)	8(8.5)
**Sedentary behaviour recommendations**								
Screen time	172(68.8)	107(42.8)	93(37.2)	68(27.2)	79(84.0)	72(76.6)	75(79.8)	58(61.7)
Reading time	146(58.4)	214(85.6)	229(91.6)	139(55.6)	41(43.6)	51(54.3)	57(60.6)	25(26.6)
Restrained time bouts	-	-	-	-	28(29.8)	33(35.1)	37(39.4)	5(5.3)
Sedentary behaviour definition 1	99(39.6)	94(37.6)	83(33.2)	42(16.8)	33(35.1)	37(39.4)	44(46.8)	14(14.9)
Sedentary behaviour definition 2	-	-	-	-	14(14.9)	17(18.1)	14(14.9)	0
**Sleep recommendation**								
Total sleep time	123(49.2)	184(73.6)	201(80.4)	86(34.4)	34(36.2)	84(89.4)	76(80.9)	26(27.7)
**Overall guidelines**								
Physical activity + sedentary behaviour definition 1 + sleep	31(12.4)	45(18.0)	69(27.6)	5(2.0)	2(2.1)	16(17.0)	21(22.3)	0
Physical activity + sedentary behaviour definition 2 + sleep	-	-	-	-	1(1.1)	9(9.6)	7(7.4)	0

Sedentary behaviour definition 1: screen time + reading time

Sedentary behaviour definition 2: screen time + reading time + restrained time

Data presented as frequency (percentage) for all variables

The tracking of recommendation and overall guideline adherence across 2, 4, and 6 months of age is shown in Table 3. In the questionnaire sample, infants that met a recommendation or the overall guidelines at 2 months, compared to those who did not, were 1.8–8.2 times significantly more likely to meet that recommendation or the overall guidelines at subsequent time points. Similar findings were observed for the time-use diary sub-sample, except meeting the restrained time and sleep time recommendations as well as the overall guidelines did not significantly track across time-points.

**Table 3:** Tracking of recommendation and overall guideline adherence across 2, 4, and 6 months of age time-points in the questionnaire and time-use diary samples

	Questionnaire		Time-use diary	
	OR(95%CI)^a^	*P* value	OR(95%CI)^a^	*P* value
**Physical activity recommendation**				
Tummy time	**3.21(1.99,5.20)**	** < 0.001**	**2.26(1.00,5.09)**	**0.049**
**Sedentary behaviour recommendations**				
Screen time	**6.13(3.45,10.89)**	** < 0.001**	**4.25(1.58,11.34)**	**0.004**
Reading time	**8.23(3.37,20.12)**	** < 0.001**	**4.04(1.95,8.39)**	** < 0.001**
Restrained time bouts	-	-	1.56(0.84,2.90)	0.162
Sedentary behaviour definition 1	**4.75(2.96,7.62)**	** < 0.001**	**3.36(1.65,6.87)**	** < 0.001**
Sedentary behaviour definition 2	-	-	**2.38(1.07,5.32)**	**0.034**
**Sleep Recommendation**				
Sleep time	**1.78(1.13,2.80)**	**0.013**	1.50(0.60,3.77)	0.389
**Overall guidelines**				
Physical activity + sedentary behaviour definition 1 + sleep	**1.90(1.01,3.58)**	**0.047**	1.37(0.21,9.09)	0.744
Physical activity + sedentary behaviour definition 2 + sleep	-	-	-^b^	-^b^

Sedentary behaviour definition 1: screen time + reading time

Sedentary behaviour definition 2: screen time + reading time + restrained time bouts

*OR* Odds ratio

^a^The odds ratio can be interpreted as the likelihood of meeting the recommendation/overall guidelines at subsequent time points (4 and 6 months of age) if the recommendation/overall guideline was met at 2 months of age

^b^The model cannot be conducted due to limited number of participants met the recommendations at T1 (*n* = 1)

The associations between recommendation and overall guideline adherence over time and ASQ-3 outcomes over time for the questionnaire and time-use diary samples provided in Tables 4 and 5. A higher number of total recommendations met across behaviours (physical activity + sedentary behaviour definition 1 + sleep) and time points (possible range: 0–9), was significantly associated with a higher gross motor development score for both questionnaire (B = 0.54; 95%CI: 0.05,1.03) and time-use diary (B = 1.16; 95%CI: 0.30,2.02) samples. A similar finding was observed in the time-use diary sub-sample when the sedentary behaviour definition 2 (screen time + reading time + restrained time) was used (B = 1.21; 95%CI: 0.26,2.17). Additionally, for the time-use diary sub-sample, meeting the overall guidelines (physical activity + sedentary behaviour definition 1 + sleep) at more time points (possible range 0–3), was associated with higher gross motor (B = 2.86; 95%CI: 0.79,4.93), fine motor (B = 2.84; 95%CI: 0.08,5.61), and problem solving (B = 3.22; 95%CI: 0.60,5.84) development scores. A similar finding was observed for the problem-solving development score when the sedentary behaviour definition 2 (screen time + reading time + restrained time) was used (B = 4.11; 95%CI: 0.11,8.10). No other associations were observed between meeting the overall guidelines and ASQ-3 outcomes. However, some associations were observed with individual recommendation adherence and ASQ-3 outcomes, as displayed in Tables 4 and 5. When categorical adherence variables were used (see Tables S1 and S2), similar patterns of associations were observed for the tummy time recommendation. Through findings were less pronounced for the reading time recommendation in the questionnaire sample and more pronounced for the sedentary behaviour definition 1 recommendation in the time-use diary sub-sample.

**Table 4:** Associations between recommendation and overall guideline adherence over time and ASQ scores over time among infants in the questionnaire sample

Number of times a recommendation or the overall guidelines is met across the three time points	Communication		Fine motor		Gross motor		Personal-social		Problem solving		Total	
	B (95%CI)	*P* value	B (95%CI)	*P* value	B (95%CI)	*P* value	B (95%CI)	*P* value	B (95%CI)	*P* value	B (95%CI)	*P* value
**Physical activity recommendation**												
Tummy time [actual range: 0–3]	0.24 (-0.65,1.13)	0.596	0.94 (-0.12,2.00)	0.082	**2.19 (1.29,3.10)**	** < 0.001**	**1.11 (0.17,2.05)**	**0.020**	0.81 (-0.28,1.91)	0.145	**5.63 (1.83,9.43)**	**0.004**
**Sedentary behaviour recommendation**												
Screen time [actual range: 0–3]	-0.13 (-0.85,0.58)	0.713	0.57 (-0.29,1.42)	0.195	0.12 (-0.64,0.88)	0.758	0.40 (-0.37,1.16)	0.307	0.42 (-0.46,1.31)	0.348	1.50 (-1.61,4.61)	0.344
Reading time [actual range:0–3]	0.59 (-0.38,1.55)	0.234	**1.23 (0.08,2.39)**	**0.036**	**1.47 (0.46,2.49)**	**0.005**	**1.38 (0.36,2.39)**	**0.008**	**1.60 (0.42,2.78)**	**0.008**	**6.45 (2.31,10.58)**	**0.002**
Sedentary behaviour definition 1 [actual range:0–3]	0.03 (-0.69,0.76)	0.932	0.50 (-0.37,1.36)	0.261	0.48 (-0.28,1.25)	0.215	0.52 (-0.25,1.28)	0.185	0.62 (-0.27,1.51)	0.172	2.30 (-0.83,5.42)	0.149
**Sleep recommendation**												
Sleep time [actual range: 0–3]	-0.73 (-1.63,0.16)	0.106	-1.01 (-2.08,0.07)	0.066	**-0.96 (-1.91,-0.01)**	**0.049**	-0.27 (-1.23,0.69)	0.579	-0.62 (-1.73,0.49)	0.272	**-3.96 (-7.83,-0.08)**	**0.045**
**Overall Guidelines**												
Physical activity + sedentary behaviour definition 1 + sleep [actual range: 0–3]	-0.17 (-1.18,0.84)	0.744	0.64 (-0.57,1.85)	0.297	0.88 (-0.19,1.95)	0.105	0.97 (-0.09,2.04)	0.074	0.87 (-0.37,2.11)	0.170	3.44 (-0.92,7.81)	0.122
**Total number of recommendations**												
Physical activity + sedentary behaviour definition 1 + sleep [actual range: 2–9]	-0.12 (-0.59,0.34)	0.607	0.19 (-0.37,0.75)	0.498	**0.54 (0.05,1.03)**	**0.032**	0.46 (-0.04,0.95)	0.069	0.31 (-0.26,0.89)	0.286	1.44 (-0.58,3.46)	0.162

Sedentary behaviour definition 1: screen time + reading time

Bold fonts indicate *p* < 0.05

Participants who had observations for all variables of interest were included in the analyses. In all models, time was included as a repeated and fixed effect and covariates (Baseline: infant sex, infant race/ethnicity, number of siblings, parental marital status, parental education, parental country of birth; Time varying: non-parental care time, parental age) were included as fixed effects

*B* Unstandardized beta coefficient, *CI* Confidence interval

**Table 5:** Associations between recommendation and overall guidelines adherence over time and ASQ-3 outcomes over time among infants in the time use-diary sub-sample

Number of times a recommendation or the overall guidelines is met across the three time points	Communication		Fine motor		Gross motor		Personal-social		Problem solving		Total	
	B (95%CI)	*P* value	B (95%CI)	*P* value	B (95%CI)	*P* value	B (95%CI)	*P* value	B (95%CI)	*P* value	B (95%CI)	*P* value
**Physical activity recommendation**												
Tummy time [actual range: 0–3]	0.28 (-1.42,1.98)	0.744	-0.03 (-2.10,2.04)	0.978	**2.28 (0.79,3.77)**	**0.003**	-0.51 (-1.99,0.97)	0.497	0.75 (-1.22,2.72)	0.452	2.54 (-4.24,9.33)	0.458
**Sedentary behaviour recommendations**												
Screen time [actual range: 0–3]	0.81 (-1.01,2.6)	0.379	1.95 (-0.22,4.12)	0.077	0.76 (-0.91,2.44)	0.366	0.10 (-1.48,1.69)	0.896	1.66 (-0.42,3.75)	0.117	5.25 (-1.92,12.42)	0.149
Reading time [actual range:0–3]	**-1.32 (-2.56,-0.09)**	**0.036**	-0.03 (-1.57,1.51)	0.972	0.60 (-0.56,1.77)	0.306	0.40 (-0.70,1.51)	0.468	1.01 (-0.46,2.48)	0.175	0.47 (-4.61,5.55)	0.855
Restrained time bouts [actual range: 0–3]	-0.09 (-1.77,1.59)	0.913	0.71 (-1.33,2.76)	0.490	0.93 (-0.62,2.47)	0.235	0.34 (-1.13,1.82)	0.643	0.25 (-1.70,2.21)	0.798	3.10 (-3.62,9.82)	0.361
Sedentary behaviour definition 1 [actual range:0–3]	-0.64 (-1.98,0.71)	0.348	0.89 (-0.74,2.52)	0.278	0.89 (-0.34,2.13)	0.154	0.46 (-0.71,1.63)	0.436	1.37 (-0.18,2.92)	0.083	2.90 (-2.47,8.28)	0.285
Sedentary behaviour definition 2 [actual range:0–3]	-0.33 (-2.41,1.74)	0.750	2.22 (-0.26,4.71)	0.079	1.42 (-0.48,3.31)	0.140	0.89 (-0.92,2.69)	0.331	2.01 (-0.38,4.39)	0.098	7.16 (-1.02,15.35)	0.085
**Sleep recommendation**												
Sleep time [range: 0–3]	-1.02 (-2.98,0.93)	0.301	1.43 (-0.95,3.82)	0.234	-0.17 (-2.00,1.65)	0.850	0.42 (-1.30,2.14)	0.628	-0.54 (-2.83,1.75)	0.638	0.15 (-7.73,8.03)	0.970
**Overall guidelines**												
Physical activity + sedentary behaviour definition 1 + sleep [actual range: 0–3]	-0.70 (-3.03,1.62)	0.550	**2.84 (0.08,5.61)**	**0.044**	**2.86 (0.79,4.93)**	**0.007**	0.84 (-1.17,2.85)	0.410	**3.22 (0.60,5.84)**	**0.017**	8.88 (-0.22,17.97)	0.056
Physical activity + sedentary behaviour definition 2 + sleep [actual range: 0–2]	-1.46 (-4.96,2.04)	0.410	2.49 (-1.77,6.74)	0.248	3.04 (-0.15,6.22)	0.061	1.64 (-1.41,4.69)	0.288	**4.11 (0.11,8.10)**	**0.044**	9.04 (-4.94,23.03)	0.202
**Total number of recommendations**												
Physical activity + sedentary behaviour definition 1 + sleep [actual range: 1–8]	-0.48 (-1.43,0.47)	0.319	0.80 (-0.36,1.96)	0.174	**1.16 (0.30,2.02)**	**0.009**	0.18 (-0.66,1.02)	0.676	0.80 (-0.30,1.90)	0.154	2.36 (-1.45,6.17)	0.222
Physical activity + sedentary behaviour definition 2 + sleep [actual range: 1–7]	-0.32 (-1.38,0.75)	0.556	0.97 (-0.32,2.25)	0.138	**1.21 (0.26,2.17)**	**0.013**	0.14 (-0.80,1.08)	0.768	0.60 (-0.63,1.83)	0.332	2.77 (-1.45,6.98)	0.195

Sedentary behaviour definition 1: screen time + reading time

Sedentary behaviour definition 2: screen time + reading time + restrained time bouts

Bold fonts indicate *p* < 0.05

Participants who had observations for all variables of interest were included in the analyses. In all models, time was included as a repeated and fixed effect and covariates (Baseline: infant sex, infant race/ethnicity, number of siblings, parental marital status, parental education, parental country of birth; Time varying: non-parental care time, parental age) were included as fixed effects

*B* Unstandardized beta coefficient, *CI* Confidence interval

The associations between recommendation and overall guideline adherence over time and milestone age outcomes for the questionnaire sample is provided in Table 6. Due to missing milestone age data, sample sizes for this analysis ranged from 202 to 216 infants for the questionnaire sample. These analyses were not conducted in the time-use diary sub-sample due to the small sample sizes (*n* = 86–92). Overall guideline adherence was not significantly associated with gross motor milestone outcomes. However, each additional time-point the physical activity recommendation was met was associated with a 5–11-day earlier acquisition of independent sitting (B = -5.33; 95%CI:-9.45,-1.21), crawling (B = -11.19; 95%CI:-17.83,-4.56), and independent standing (B = -10.59; 95%CI:-19.14,-2.04) milestones. No other associations were observed in either sample. Sensitivity analyses where exact milestone acquisition dates were reported (*n* = 185–204) produced similar findings (data not shown). Similar findings were also observed when the categorical adherence variables were used, as displayed in Table S3.

**Table 6:** Associations between recommendation adherence and overall guideline adherence over time and milestone age outcomes among infants in the questionnaire sample

Number of times a recommendation or the overall guidelines is met across the three time points	Independent sitting (days; *n* = 202)		Crawling (days; *n* = 203)		Assisted standing (days; *n* = 205)		Assisted walking (days; *n* = 205)		Independent standing (days; *n* = 206)		Independent walking (days; *n* = 216)	
	B (95%CI)	*P* value	B (95%CI)	*P*value	B (95%CI)	*P* value	OR (95%CI)	*P* value	B (95%CI)	*P* value	B (95%CI)	*P* value
**Physical activity recommendation**												
Tummy time [actual range: 0–3]	**-5.33 (-9.45,-1.21)**	**0.011**	**-11.19 (-17.83,-4.56)**	**0.001**	-5.31 (-11.89,1.28)	0.113	-6.16 (-13.78,1.76)	0.112	**-10.59 (-19.14,-2.04)**	**0.015**	-8.76 (-17.81,0.29)	0.058
**Sedentary behaviour recommendations**												
Screen time [actual range: 0–3]	0.09 (-3.47,3.65)	0.959	1.84 (-3.74,7.42)	0.516	1.54 (-3.95,7.02)	0.581	2.73 (-3.49,8.94)	0.388	3.59 (-3.52,10.70)	0.321	0.07 (-7.51,7.65)	0.986
Reading time [actual range:0–3]	0.47 (-4.79,5.74)	0.860	-2.00 (-10.29,6.30)	0.636	-2.82 (-10.87,5.23)	0.491	-4.53 (-13.69,4.63)	0.331	-3.56 (-13.71,6.59)	0.490	1.23 (-9.46,11.92)	0.821
Sedentary behaviour definition 1 [actual range:0–3]	-0.28 (-3.96,3.39)	0.879	2.17 (-3.50,7.84)	0.451	1.03 (-4.63,6.69)	0.721	0.78 (-5.61,7.17)	0.810	3.49 (-3.76,10.74)	0.344	3.08 (-4.63,10.79)	0.432
**Sleep recommendation**												
Sleep time [actual range: 0–3]	2.54 (-1.94,7.01)	0.265	1.13 (-6.17,8.44)	0.760	1.85 (-5.11,8.81)	0.600	1.80 (-6.37,9.96)	0.664	4.35 (-4.89,13.58)	0.354	5.07 (-4.52,14.65)	0.298
**Overall guidelines**												
Physical activity + Sedentary behaviour definition 1 + sleep [actual range: 0–3]	-1.19 (-6.27,3.89)	0.645	1.85 (-6.19,9.88)	0.651	-1.64 (-9.70,6.42)	0.688	0.81 (-8.23,9.85)	0.861	2.28 (-7.89,12.44)	0.659	3.14 (-7.73,14.00)	0.570
**Total number of recommendations**												
Physical activity + sedentary behaviour definition 1 + sleep [actual range: 2–9]	-1.06 (-3.37,1.24)	0.364	-2.06 (-5.73,1.62)	0.271	-0.68 (-4.35,2.99)	0.714	-1.01 (-5.16,3.14)	0.631	-0.52 (-5.19,4.16)	0.827	0.03 (-4.91,4.98)	0.989

Sedentary behaviour definition 1: screen time + reading time

Bold fonts indicate *p* < 0.05

Participants who had observations for all variables of interest were included in the analyses. Covariates (Baseline: child sex, race/ethnicity, the number of siblings, parental age, parental marital status, parental education, parental country of birth; Average across time points: non-parental care time) were included in all models

*B* Unstandardized beta coefficient, *CI* Confidence interval

Associations between recommendation and overall guideline adherence over time and AIMS outcomes among infants in the time-use diary sub-sample are shown in Table 7. A higher total number of recommendations met across behaviours (physical activity + sedentary behaviour definition 1 + sleep) and time points was associated with a higher total (B = 0.97; 95%CI: 0.20,1.74) and percentile (B = 5.02; 95%CI: 1.25,8.79) AIMS score. Similar findings were observed when the sedentary behaviour definition 2 (screen time + reading time + restrained time) was used (total: B = 0.97, 95%CI: 0.09,1.85; percentile: B = 4.86, 95%CI: 0.55,9.17). Meeting the overall guidelines was also associated with a higher total (physical activity + sedentary behaviour definition 1 + sleep: B = 2.36; 95%CI: 0.50,4.22 and physical activity + sedentary behaviour definition 2 + sleep: B = 3.45; 95%CI: 0.28,6.63) and percentile (physical activity + sedentary behaviour definition 1 + sleep: B = 12.28; 95%CI: 3.17,21.40 and physical activity + sedentary behaviour definition 2 + sleep: B = 16.67; 95%CI: 1.03,32.30) AIMS score, regardless of what sedentary behaviour definition was used. Few associations were observed with individual recommendation adherence and AIMS outcomes, as displayed in Tables 7 and S4.

**Table 7:** Associations between recommendation and overall guideline adherence over time and AIMS outcomes among infants in the time-use diary sub-sample

Number of times a recommendation or the overall guidelines is met across the three time points	AIMS prone		AIMS supine		AIMS sit		AIMS stand^a^		AIMS total		AIMS percentile	
	B (95%CI)	*P* value	B (95%CI)	*P* value	B (95%CI)	*P* value	OR (95%CI)	*P* value	B (95%CI)	*P* value	B (95%CI)	*P* value
**Physical activity recommendation**												
Tummy time [actual range: 0–3]	0.64 (-0.08,1.36)	0.083	**0.29 (0.02,0.59)**	**0.048**	-0.18 (-0.72,0.36)	0.503	1.37 (0.79,2.40)	0.266	0.83 (-0.57,2.21)	0.238	6.09 (-0.63,12.82)	0.075
**Sedentary behaviour recommendations**												
Screen time [actual range: 0–3]	**0.79 (0.03,1.56)**	**0.046**	0.25 (-0.07,0.57)	0.117	-0.12 (-0.70,0.47)	0.687	1.21 (0.69,2.14)	0.508	0.98 (-0.51,2.47)	0.196	5.38 (-1.94,12.70)	0.148
Reading time [actual range:0–3]	-0.05 (-0.59,0.50)	0.869	0.06 (-0.16,0.28)	0.574	0.19 (-0.21,0.59)	0.358	1.32 (0.88,1.98)	0.183	0.26 (-0.77,1.30)	0.614	0.93 (-4.16,6.01)	0.718
Restrained time bouts [actual range: 0–3]	0.32 (-0.42,1.05)	0.388	-0.06 (-0.36,0.24)	0.712	0.39 (-0.15,0.92)	0.153	1.54 (0.88,2.70)	0.131	0.74 (-0.65,2.12)	0.293	2.94 (-3.90,9.77)	0.395
Sedentary behaviour 1 [actual range:0–3]	0.32 (-0.27,0.90)	0.281	0.20 (-0.04,0.43)	0.100	0.15 (-0.28,0.59)	0.479	1.32 (0.85,2.06)	0.213	0.73 (-0.37,1.83)	0.191	3.61 (-1.81,9.03)	0.189
Sedentary behaviour 2 [actual range: 0–3]	0.54 (-0.39,1.46)	0.252	0.15 (-0.23,0.53)	0.423	0.12 (-0.57,0.81)	0.734	1.98 (0.90,4.34)	0.089	0.94 (-0.82,2.70)	0.292	3.67 (-5.02,12.34)	0.403
**Sleep recommendation**												
Sleep time [actual range: 0–3]	0.62 (-0.18,1.42)	0.125	0.29 (-0.04,0.62)	0.079	0.27 (-0.33,0.86)	0.373	1.31 (0.73,2.34)	0.371	1.20 (-0.32,2.72)	0.121	4.35 (-3.18,11.88)	0.254
**Overall guidelines**												
Physical activity + sedentary behaviour definition 1 + sleep [actual range: 0–3]	**1.52 (0.56,2.48)**	**0.002**	**0.55 (0.16,0.95)**	**0.007**	0.12 (-0.63,0.87)	0.757	**2.85 (1.10,7.40)**	**0.0e31**	**2.36 (0.50,4.22)**	**0.014**	**12.28 (3.17,21.40)**	**0.009**
Physical activity + sedentary behaviour definition 2 + sleep [actual range: 0–2]	**2.13 (0.48,3.78)**	**0.012**	**0.75 (0.07,1.43)**	**0.031**	0.39 (-0.87,1.66)	0.540	3.13 (0.70,13.89)	0.134	**3.45 (0.28,6.63)**	**0.033**	**16.67 (1.03,32.30)**	**0.037**
**Total number of recommendations**												
Physical activity + sedentary behaviour definition 1 + sleep [actual range: 1–8]	**0.54 (0.14,0.94)**	**0.009**	**0.28 (0.12,0.44)**	** < 0.001**	0.09 (-0.22,0.40)	0.559	**1.40 (1.01,1.97)**	**0.050**	**0.97 (0.20,1.74)**	**0.014**	**5.02 (1.25,8.79)**	**0.010**
Physical activity + sedentary behaviour definition 2 + sleep [actual range: 1–7]	**0.60 (0.14,1.05)**	**0.011**	**0.26 (0.07,0.44)**	**0.007**	0.04 (-0.31,0.39)	0.805	**1.48 (1.01,2.19)**	**0.042**	**0.97 (0.09,1.85)**	**0.031**	**4.86 (0.55,9.17)**	**0.028**

Sedentary behaviour definition 1: screen time + reading time

Sedentary behaviour definition 2: screen time + reading time + restrained time bouts

Bold fonts indicate *p* < 0.05

Participants who had observations for all variables of interest were included in the analyses. Covariates (AIMS assessment: infant age; Baseline: infant sex, infant race/ethnicity, number of siblings, parental age, parental marital status, parental education, parental country of birth; Average across time points: non-parental care time) were included in all models. One participant had missing data on AIMS

Mean imputations were conducted for 6 missing data on average non-parental care time

*B* Unstandardized beta coefficient, *OR* Odds ratio, *CI* Confidence interval, *AIMS* Alberta Infant Motor Scale

^a^Due to distribution, AIMS stand was dichotomised as a dummy variable (value = 0 reference]: score = 2; value = 1: score > 2)

## Discussion

This study filled a critical gap in the literature by examining adherence to the 24-Hour Movement Guidelines and associations between guideline adherence over time and various domains of development in a relatively diverse sample of infants. Study objectives were comprehensively addressed by utilizing a sample with questionnaire measures as well as a sub-sample with more precise time-use diary measures of movement behaviours and directly observed gross motor development. Overall, few infants met the 24-Hour Movement Guidelines across the first 6 months of life, and meeting individual recommendations as well as the overall guidelines appeared to track over time. Additionally, when considering the different measures and samples, meeting more movement behaviour recommendations over the first 6 months of life was consistently associated with more advanced gross motor development.

The low adherence to 24-Hour Movement Guidelines in this sample of infants is consistent with another regional sample of infants from Australia that had a mean age of 3.6 months. [13] Specifically, only 4% of infants in the Australia sample met the overall guidelines, including physical activity (≥ 30 min/day of tummy time), sedentary behaviour (no screen time and restrained time < 1 h), and sleep recommendations (14–17 h for 0–3 month olds or 12–16 h for 4–11 month olds) [13]. Similarly, adherence appears low in other pediatric age groups in Canada [7, 8] and internationally [9]. Specifically, in a regional sample of toddlers from Edmonton, Canada that were recruited using similar procedures as the present study, only 15% met the overall guidelines [8]. In other regional samples of toddlers from New Zealand and Australia, 0.6–9% were reported to meet the overall guidelines [16, 22]. Meta-analysis findings in samples of preschoolers, children, and adolescence across 23 countries, including Canada, indicate guideline adherence is 11%, 10%, and 3%, respectively. The low guideline adherence across countries and pediatric age groups, beginning right from the start of life, is a concerning public health issue that may benefit from early intervention.

A novel aspect of this study is the longitudinal study design, which enables the examination of guideline adherence over time. Though previous research has shown that physical activity and sedentary behaviour in early childhood can track over time [37, 38], minimal evidence exists on whether 24-Hour Movement Guideline adherence tracks over time. In the meta-analysis previously discussed, only eight out of 63 included studies were longitudinal, including three in the preschool-aged group [10–12]. Of these eight studies, only two examined the tracking of guideline adherence [22, 39]. Specifically, in a regional sample of children from New Zealand, meeting the overall guidelines at age 1 and 2 was associated with a higher likelihood of meeting the overall guidelines at age 2 and 5, respectively [22]. Though meeting the physical activity and sleep recommendations at age 1 was not significantly associated with meeting these recommendations at age 2 [22]. Additionally, approximately half of a regional sample of children from Canada, who were 9 to 11 years of age at baseline, were categorized as compliers or non-compliers to 24-Hour Movement Guidelines over an 8-year period [39]. Our findings suggest that even at a very young age, behavioural patterns can be formed that persist over time. However, our study only looked at movement behaviours in the first 6 months of life. Therefore, future research should examine if behavioural patterns in infancy track beyond this age.

The development of 24-Hour Movement Guidelines across the lifespan in Canada and for specific age groups internationally has resulted in a number of studies examining how meeting individual recommendations and the overall guidelines impacts health [15]. Specifically, in a systematic review published in 2020, 31 studies from 21 different countries were included that examined the association between meeting guidelines and health indicators across the lifespan [15]. However, in terms of the early years, minimal evidence was identified especially for health indicators other than adiposity [15], highlighting the importance of the present study. Specifically, it was concluded in the review that meeting individual recommendations and/or the overall guidelines was not associated with adiposity in toddlers, based on findings from two studies [8, 16], or consistently associated with adiposity in preschoolers, based on findings from five studies [7, 17, 20–22]. Similarly, in the only included study with an infant sample, no associations were observed between meeting individual recommendations or overall guidelines and adiposity [13]. In terms of other health indicators, some associations were observed in preschoolers between meeting individual recommendations and/or the overall guidelines and lower behavioural and emotional problems [14], higher quality of life [18], and more advanced social-cognitive development [19]. Though it was noted in the review that these findings were preliminary as each outcome was only examined in one study [15]. A study published after the review, which utilized the same sample of New Zealanders as another included study, found inconsistent associations between guideline adherence at age 1 and 2 and psychosocial functioning at age 5 [40]. Therefore, the findings of the present study make an important contribution to the literature by providing evidence that guidelines adherence in the first 6 months of life may be important for gross motor development, an health indicator not found in the above mentioned systematic review for early years children [15]. Though findings in the present study for other developmental areas, which have some overlap with the social-emotional indicators of previous work in toddlers and preschoolers [14, 19, 40], were inconsistent. Future research is needed to not only better understand the short-term health implications of meeting 24-Hour Movement Guidelines in infancy but also the longer-term health implications.

Another novel aspect of our study was the inclusion of a sub-sample with more precise measures. Consistency in findings across questionnaire and time-use diary samples provides more confidence in study findings. For the prevalence of guideline adherence across all 3 time points, similar findings were observed between questionnaire and time-use diary samples for sedentary behaviour (definition 1) and sleep recommendations as well as the overall guidelines. However, a larger difference was observed for the physical activity recommendation pertaining to tummy time. For associations between guideline adherence and ASQ-3 outcomes, which were examined in both questionnaire and time-use diary samples, meeting more tummy time recommendations and total number of recommendations across time-points were consistently associated with more advanced gross motor development. As outlined in the methods section of the present paper, the validity of the questionnaire and time-use diary measures of tummy time against a GENEActiv accelerometer measure have previously been examined in a subsample of Early Movers participants [31]. The relative concurrent validity between subjective and device-based measures was found to be strong suggesting they are likely appropriate measures for examining the association between tummy time and health indicators, such as gross motor development [31]. However, in regard to absolute validity the time-use diary measure was found to provide a more precise estimate of tummy time compared to the questionnaire, which tended to overestimate tummy time at the individual level [31]. In particular, it was noted that the time-use diary measure was most accurate when classifying individuals as meeting versus not meeting the tummy time recommendation (i.e., > 30 min/day). Therefore, it was concluded in the validity study that the time-use diary measure may be more appropriate for prevalence studies. Overall, this suggests that the lower adherence rates observed for tummy time in the time-use diary sub-sample compared to the questionnaire sample may be a more accurate reflection of prevalence in this age group.

This study has a number of strengths, including the focus on infants, the longitudinal study design, the relatively diverse sample, the comprehensive assessment of movement behaviours using both questionnaires as well as time-use diaries in a sub-sample, the adjustment for key infant and parental covariates, and the inclusion of outcomes that span different developmental domains. Some study limitations also warrant acknowledging. For instance, despite the use of multiple measures of movement behaviours with acceptable psychometric properties, the questionnaire and time-use diary measures were still subjective measures and therefore more prone to recall and social desirability bias. Based on our analysis, a number of participants were excluded due to incomplete data at one or more time points, which resulted in demographic differences between included and excluded participants. Therefore, the generalizability of findings needs to be interpreted with caution. Additionally, residual confounding may still have occurred, despite the adjustment for key covariates. Also, given the large number of models run to address the study objectives, there was an increased risk of type 1 error. As a result, we tried to focus on the patterns and trends of the findings. Finally, given the sample size and the low adherence observed it was not possible to examine the association between different trajectories of guideline adherence and development over time.

## Conclusion

The early years is a period of rapid development and an optimal balance of physical activity, sedentary behaviour, and sleep is thought to help support optimal development in these first few years of live [1]. However, research to date on movement behaviours in the early years has primarily focused on preschoolers, with the least amount of evidence in the infant age group [9, 15, 41]. Findings from this study suggest guideline adherence is low across the first 6 months of infancy and these patterns may persist overtime. Additionally, findings suggest meeting more recommendations over this period may be important for gross motor development. Consequently, early intervention targeting parents and caregivers with guideline dissemination and activation strategies may help to promote healthy infant development. Further research is needed to understand the long-term implications of sub-optimal movement behaviour patterns in infancy.

## Supplementary Information

**Additional file 1: Supplementary Table 1.** Associations between consistent recommendation adherence and ASQ scores over time among infants in the questionnaire sample. **Supplementary Table 2.** Associations between consistent recommendation adherence and ASQ scores over time among infants in the time-use diary sample. **Supplementary Table 3. **Associations between consistent recommendation adherence and milestone age outcomes among infants in the questionnaire sample. **Supplementary Table 4.** Associations between consistent recommendation adherence and AIMS outcomes among infants in the time-use diary sub-sample.

## Data Availability

The dataset generated and analysed during the current study are not publicly available due ethical restrictions but are available from the corresponding author on reasonable request.

## Abbreviations

AIMS: Alberta Infant Motor Scale
ASQ-3: Ages & Stages Questionnaire
B: Unstandardized beta coefficient
CI: Confidence interval
GEE: Generalized estimating equations
ICC: Intraclass correlation coefficient
OR: Odds ratio
WHO: World Health Organization
