# Supplementary material for: Adherence to Canadian 24-Hour Movement Guidelines among infants and associations with development: a longitudinal study
Source: Int J Behav Nutr Phys Act. 2022 Dec 15;19:154. doi: 10.1186/s12966-022-01397-8 (PMC9753321; doi:10.1186/s12966-022-01397-8)
Supplement: Supplementary file 1 — Additional file 1: Supplementary Table 1. Associations between consistent recommendation adherence and ASQ scores over time among infants in the questionnaire sample. Supplementary Table 2. Associations between consistent recommendation adherence and ASQ scores over time among infants in the time-use diary sample. Supplementary Table 3. Associations between consistent recommendation adherence and milestone age outcomes among infants in the questionnaire sample. Supplementary Table 4. Associations between consistent recommendation adherence and AIMS outcomes among infants in the time-use diary sub-sample. [file 12966_2022_1397_MOESM1_ESM.docx]

**Table S1**. Associations between consistent recommendation adherence and ASQ scores over time among infants in the questionnaire sample

| Meeting a recommendation at all 3 times vs. not meeting the recommendation at all 3 times | Communication | | Fine motor | | Gross motor | | Personal-social | | Problem solving | | Total | |
| --- | --- | --- | --- | --- | --- | --- | --- | --- | --- | --- | --- | --- |
|  | B (95%CI) | P value | B (95%CI) | P value | B (95%CI) | P value | B (95%CI) | P value | B (95%CI) | P value | B (95%CI) | P value |
| **Physical activity recommendation** | | | | | | | | | | | | |
| Tummy time Met | 0.14  (-1.44,1.72) | 0.863 | 1.55  (-0.34,3.44) | 0.108 | **3.24**  **(1.60,4.87)** | **<0.001** | **2.23**  **(0.57,3.90)** | **0.009** | 1.79  (-0.16,3.73) | 0.071 | **9.74**  **(2.97,16.51)** | **0.005** |
| Not Met | Reference | | Reference | | Reference | | Reference | | Reference | | Reference | |
| **Sedentary behaviour recommendation** | | | | | | | | | | | | |
| Screen time Met | 0.25  (-1.50,2.01) | 0.775 | 1.51  (-0.59,3.62) | 0.157 | 0.88  (-0.99,2.75) | 0.356 | 0.94  (-0.93,2.81) | 0.321 | 1.70  (-0.46,3.86) | 0.123 | 6.20  (-1.40,13.80) | 0.109 |
| Not Met | Reference | | Reference | | Reference | | Reference | | Reference | | Reference | |
| Reading time Met | 0.43  (-1.21,2.06) | 0.609 | 0.63  (-1.33,2.59) | 0.528 | **1.99**  **(0.27,3.72)** | **0.024** | 1.29  (-0.44,3.02) | 0.144 | 1.58  (-0.43,3.59) | 0.123 | 6.24  (-0.83,13.31) | 0.083 |
| Not Met | Reference | | Reference | | Reference | | Reference | | Reference | | Reference | |
| Sedentary behaviour definition 1 Met | -0.36  (-2.47,1.76) | 0.741 | 1.02  (-1.52,3.56) | 0.431 | 2.12  (-0.12,4.36) | 0.063 | 1.00  (-1.25,3.26) | 0.380 | 1.46  (-1.15,4.06) | 0.272 | 6.10  (-3.06,15.27) | 0.191 |
| Not Met | Reference | | Reference | | Reference | | Reference | | Reference | | Reference | |
| **Sleep recommendation** | | | | | | | | | | | | |
| Sleep time Met | -0.40  (-2.02,1.21) | 0.623 | -1.35  (-3.29,0.59) | 0.171 | -1.16  (-2.88,0.56) | 0.187 | 0.31  (-1.42,2.03) | 0.727 | -0.87  (-2.87,1.13) | 0.392 | -4.25  (-11.27,2.78) | 0.235 |
| Not Met | Reference | | Reference | | Reference | | Reference | | Reference | | Reference | |
| **Overall Guidelines** | | | | | | | | | | | | |
| Physical activity + sedentary behaviour definition 1 +sleep Met | - | - | - | - | - | - | - | - | - | - | - | - |
| Not Met | Reference | | Reference | | Reference | | Reference | | Reference | | Reference | |

Abbreviations: B, unstandardized beta coefficient; CI, confidence interval.

Sedentary behaviour definition 1: screen time + reading time

**Bold fonts** indicate p<0.05.

Note: Participants who had three observations on variables of interest were included in the analyses. In all models, time was included as a repeated and fixed effect and covariates (Baseline: infant sex, infant race/ethnicity, number of siblings, parental marital status, parental education, parental country of birth; Time varying: non-parental care time, parental age) were included as fixed effects.

Note: The analysis for meeting the overall guidelines was not performed because only 2% of the sample met the overall guidelines at all three time points.

**Table S2**. Associations between consistent recommendation adherence and ASQ scores over time among infants in the time-use diary sample

| Meeting a recommendation at all 3 times vs. not meeting the recommendation at all 3 times | Communication | | Fine motor | | Gross motor | | Personal-social | | Problem solving | | Total | |
| --- | --- | --- | --- | --- | --- | --- | --- | --- | --- | --- | --- | --- |
|  | B (95%CI) | P value | B (95%CI) | P value | B (95%CI) | P value | B (95%CI) | P value | B (95%CI) | P value | B (95%CI) | P value |
| **Physical activity recommendation** | | | | | | | | | | | | |
| Tummy time Met | -0.51  (-4.38,3.36) | 0.795 | -3.62  (-8.13,0.89) | 0.115 | **3.63**  **(0.10,7.16)** | **0.044** | -2.81  (-6.46,0.64) | 0.130 | -0.22  (-4.68,4.24) | 0.923 | -3.01  (-16.48,10.45) | 0.660 |
| Not Met | Reference | | Reference | | Reference | | Reference | | Reference | | Reference | |
| **Sedentary behaviour recommendation** | | | | | | | | | | | | |
| Screen time Met | 1.07  (-1.36,3.50) | 0.387 | 1.95  (-0.89,4.79) | 0.178 | 1.06  (-1.17,3.30) | 0.349 | -0.48  (-2.77,1.81) | 0.681 | 1.83  (-0.96,4.63) | 0.197 | 5.43  (-3.02,13.88) | 0.207 |
| Not Met | Reference | | Reference | | Reference | | Reference | | Reference | | Reference | |
| Reading time Met | -1.08  (-3.50,1.34) | 0.381 | 1.51  (-1.33,4.35) | 0.297 | **2.32**  **(0.10,4.53)** | **0.040** | 2.26  (-0.02,4.54) | 0.052 | 2.61  (-0.16,5.37) | 0.065 | 7.45  (-0.94,15.85) | 0.082 |
| Not Met | Reference | | Reference | | Reference | | Reference | | Reference | | Reference | |
| Restrained time Met | 0.47  (-4.84,5.79) | 0.861 | -1.79  (-8.05,4.47) | 0.574 | 0.06  (-4.82,4.94) | 0.981 | -0.47  (-5.57,4.62) | 0.855 | 0.46  (-5.63,6.55) | 0.883 | -1.18  (-19.69,17.34) | 0.901 |
| Not Met | Reference | Reference | Reference | Reference | Reference | Reference |  |  |  |  |  |  |
| Sedentary behaviour definition 1 Met | 0.25  (-2.82,3.32) | 0.872 | **3.92**  **(0.34,7.49)** | **0.032** | 2.64  (-0.17,5.46) | 0.066 | 1.24  (-1.67,4.15) | 0.402 | **4.84**  **(1.36,8.32)** | **0.007** | **13.48**  **(2.89,24.07)** | **0.013** |
| Not Met | Reference | | Reference | | Reference | | Reference | | Reference | | Reference | |
| Sedentary behaviour definition 2 Met | - | - | - | - | - | - | - | - | - | - | - | - |
| Not Met | Reference | | Reference | | Reference | | Reference | | Reference | | Reference | |
| **Sleep recommendation** | | | | | | | | | | | | |
| Sleep time Met | -2.21  (-4.74,0.32) | 0.087 | **3.36**  **(0.39,6.34)** | **0.027** | 0.50  (-1.84,2.84) | 0.674 | 1.55  (-0.86,3.96) | 0.208 | -1.80  (-4.71,1.11) | 0.225 | 1.39  (-7.48,10.26) | 0.758 |
| Not Met | Reference | | Reference | | Reference | | Reference | | Reference | | Reference | |
| **Overall Guidelines** | | | | | | | | | | | | |
| Physical activity + sedentary behaviour definition 1 +sleep Met | - | - | - | - | - | - | - | - | - | - | - | - |
| Not Met | Reference | | Reference | | Reference | | Reference | | Reference | | Reference | |
| Physical activity + sedentary behaviour definition 2 +sleep Met | - | - | - | - | - | - | - | - | - | - | - | - |
| Not Met | Reference | | Reference | | Reference | | Reference | | Reference | | Reference | |

Abbreviations: B, unstandardized beta coefficient; CI, confidence interval.

Sedentary behaviour definition 1: screen time + reading time

**Bold fonts** indicate p<0.05.

Note: Participants who had three observations on variables of interest were included in the analyses. In all models, time was included as a repeated and fixed effect and covariates (Baseline: infant sex, infant race/ethnicity, number of siblings, parental marital status, parental education, parental country of birth; Time varying: non-parental care time, parental age) were included as fixed effects.

Note: Analyses for meeting the sedentary behaviour definition 2 recommendation and the overall guidelines were not performed because 0% of the sample met the recommendation or overall guidelines at all three time points.

**Table S3**. Associations between consistent recommendation adherence and milestone age outcomes among infants in the questionnaire sample

| Meeting a recommendation at all 3 times vs. not meeting the recommendation at all 3 times | Independent sitting (days; n=202) | | Crawling (days; n=203) | | Assisted standing  (days; n=205) | | Assisted walking  (days; n=205) | | Independent standing  (days; n=206) | | Independent walking  (days; n=216) | |
| --- | --- | --- | --- | --- | --- | --- | --- | --- | --- | --- | --- | --- |
|  | B (95%CI) | P value | B (95%CI) | P value | B (95%CI) | P value | OR (95%CI) | P value | B (95%CI) | P value | B (95%CI) | P value |
| **Physical activity recommendation** | | | | | | | | | | | | |
| Tummy time Met | -5.08  (-12.81,2.66) | 0.197 | **-13.12**  **(-25.49,-0.75)** | **0.038** | -6.94  (-18.95,5.06) | 0.255 | -11.43  (-25.23,2.38) | 0.104 | **-16.89**  **(-32.55,-1.23)** | **0.035** | -14.79  (-31.50,1.93) | 0.083 |
| Not Met | Reference | | Reference | | Reference | | Reference | | Reference | | Reference | |
| **Sedentary behaviour recommendations** | | | | | | | | | | | | |
| Screen time Met | -0.06  (-8.90,8.77) | 0.989 | -2.28  (-16.14,11.57) | 0.745 | -0.36  (-14.01,13.29) | 0.958 | -2.40  (-17.88,13.08) | 0.760 | 2.40  (-15.16,19.95) | 0.788 | -6.24  (-24.95,12.46) | 0.511 |
| Not Met | Reference | | Reference | | Reference | | Reference | | Reference | | Reference | |
| Reading time Met | -2.25  (-10.50,5.99) | 0.591 | 1.35  (-11.89,14.59) | 0.841 | 0.46  (-12.25,13.18) | 0.943 | -1.30  (-16.11,13.50) | 0.862 | 2.98  (-13.61,19.56) | 0.724 | 5.50  (-12.10,23.10) | 0.538 |
| Not Met | Reference | | Reference | | Reference | | Reference | | Reference | | Reference | |
| Sedentary behaviour definition 1 Met | -8.36  (-19.10,2.39) | 0.127 | -5.29  (-21.67,11.10) | 0.525 | -5.08  (-21.66,11.49) | 0.546 | -8.21  (-26.69,10.27) | 0.382 | 0.95  (-20.28,22.18) | 0.930 | -6.09  (-28.59,16.40) | 0.594 |
| Not Met | Reference | | Reference | | Reference | | Reference | | Reference | | Reference | |
| **Sleep recommendation** | | | | | | | | | | | | |
| Sleep time Met | -2.04  (-9.96,5.89) | 0.613 | -6.30  (-19.20,6.60) | 0.337 | -1.12  (-16.60,11.36) | 0.860 | 1.18  (-13.33,15.70) | 0.873 | 6.15  (-10.19,22.49) | 0.459 | 4.26  (-13.03,21.56) | 0.627 |
| Not Met | Reference | | Reference | | Reference | | Reference | | Reference | | Reference | |
| **Overall guidelines** | | | | | | | | | | | | |
| Physical activity+ Sedentary behaviour definition 1 +sleep Met | - | - | - | - | - | - | - | - | - | - | - | - |
| Not Met | Reference | | Reference | | Reference | | Reference | | Reference | | Reference | |

Abbreviations: B, unstandardized beta coefficient; CI, confidence interval

Sedentary behaviour definition 1: screen time + reading time

**Bold fonts** indicate p<0.05.

Note: Covariates (Baseline: child sex, race/ethnicity, the number of siblings, parental age, parental marital status, parental education, parental country of birth; Average across time points: non-parental care time) were included in all models.

Note: The analysis for meeting the overall guidelines was not performed because only 2% of the sample met the overall guidelines at all three time points.

**Table S4** Associations between consistent recommendation adherence and AIMS outcomes among infants in the time-use diary sub-sample

| Meeting a recommendation at all 3 times vs. not meeting the recommendation at all 3 times | AIMS prone | | | AIMS supine | | | AIMS sit | | | AIMS stand^1^ | | | AIMS total | | AIMS percentile | |
| --- | --- | --- | --- | --- | --- | --- | --- | --- | --- | --- | --- | --- | --- | --- | --- | --- |
|  | B (95%CI) | P value | | B (95%CI) | P value | | B (95%CI) | P value | | OR (95%CI) | P value | | B (95%CI) | P value | B (95%CI) | P value |
| **Physical activity recommendation** | | | | | | | | | | | | | | | | |
| Tummy time Met | 0.80  (-1.36,2.96) | 0.463 | | 0.26  (-0.58,1.10) | 0.535 | | 0.54  (-1.06,2.15) | 0.502 | | 4.41  (0.50,39.29) | 0.184 | | 1.83  (-2.23,5.89) | 0.372 | 13.23  (-6.78,33.25) | 0.192 |
| Not Met | Reference | | Reference | | | Reference | | | Reference | | | Reference | | | Reference | |
| **Sedentary behaviour recommendations** | | | | | | | | | | | | | | | | |
| Screen time Met | **0.43**  **(-0.95,1.81)** | **0.540** | | 0.09  (-0.44,0.63) | 0.728 | | -0.30  (-1.33,0.72) | 0.558 | | 1.63  (0.60,4.43) | 0.334 | | 0.34  (-2.26,2.95) | 0.794 | 3.37  (-9.53,16.26) | 0.605 |
| Not Met | Reference | | Reference | | | Reference | | | Reference | | | Reference | | | Reference | |
| Reading time Met | -0.22  (-1.64,1.20) | 0.755 | | -0.16  (-0.71,0.39) | 0.568 | | 0.59  (-0.46,1.64) | 0.264 | | 1.39  (0.48,4.05) | 0.543 | | 0.25  (-2.42,2.93) | 0.851 | -0.23  (-13.50,13.03) | 0.972 |
| Not Met | Reference | | Reference | | | Reference | | | Reference | | | Reference | | | Reference | |
| Restrained time bouts Met | -0.004  (-3.11,3.11) | 0.998 | | 0.03  (-1.18,1.23) | 0.968 | | 0.25  (-2.06,2.56) | 0.828 | | 4.11  (0.26,66.26) | 0.637 | | 0.55  (-5.30,6.41) | 0.852 | -0.86  (-29.88,28.17) | 0.953 |
| Not Met | Reference | | Reference | | | Reference | | | Reference | | | Reference | | | Reference | |
| Sedentary behaviour 1 Met | 0.14  (-1.72,2.00) | 0.884 | | 0.20  (-0.52,0.92) | 0.577 | | 0.10  (-1.28,1.49) | 0.881 | | 1.15  (0.29,4.64) | 0.846 | | 0.44  (-3.06,3.94) | 0.804 | 4.49  (-12.84,21.82) | 0.608 |
| Not Met | Reference | | Reference | | | Reference | | | Reference | | | Reference | | | Reference | |
| Sedentary behaviour 2 Met | **-** | **-** | | **-** | **-** | | **-** | **-** | | **-** | **-** | | **-** | **-** | **-** | **-** |
| Not Met | Reference | | Reference | | | Reference | | | Reference | | | Reference | | | Reference | |
| **Sleep recommendation** | | | | | | | | | | | | | | | | |
| Sleep time Met | 1.05  (-0.44,2.53) | 0.164 | | 0.38  (-0.20,0.95) | 0.199 | | 0.78  (-0.32,1.89) | 0.161 | | 1.36  (0.45,4.11) | 0.591 | | 2.25  (-0.53,5.04) | 0.111 | 10.25  (-3.59,24.08) | 0.144 |
| Not Met | Reference | | Reference | | | Reference | | | Reference | | | Reference | | | Reference | |
| **Overall guidelines** | | | | | | | | | | | | | | | | |
| Physical activity+ sedentary behaviour definition 1 +sleep  Met | **-** | **-** | | **-** | **-** | | **-** | **-** | | **-** | **-** | | **-** | **-** | **-** | **-** |
| Not Met | Reference | | Reference | | | Reference | | | Reference | | | Reference | | | Reference | |
| ..Physical activity + sedentary behaviour definition 2 +sleep  Met | **-** | **-** | | **-** | **-** | | **-** | **-** | | **-** | **-** | | **-** | **-** | **-** | **-** |
| Not Met | Reference | | Reference | | | Reference | | | Reference | | | Reference | | | Reference | |

Abbreviations: B, unstandardized beta coefficient; OR, odds ratio; CI, confidence interval; AIMS, Alberta Infant Motor Scale.

Sedentary behaviour definition 1: screen time + reading time

Sedentary behaviour definition 2: screen time + reading time + restrained time bouts

**Bold fonts** indicate p<0.05.

Note: Covariates (AIMS assessment: infant age; Baseline: infant sex, infant race/ethnicity, number of siblings, parental age, parental marital status, parental education, parental country of birth; Average across time points: non-parental care time) were included in all models. One participant had missing data on AIMS

^1^Due to distribution, AIMS stand was dichotomised as a dummy variable (value = 0 reference]: score =2; value = 1: score >2)

Mean imputations were conducted for 6 missing data on average non-parental care time.

Note: Analyses for meeting the sedentary behaviour definition 2 recommendation and the overall guidelines were not performed because 0% of the sample met the recommendation or overall guidelines at all three time points.
